# Self-Reported Medical Errors and Primary Care Physicians’ Performance and Confidence in Delivering Care: A Multilevel Empirical Study in China

**DOI:** 10.3390/healthcare13040360

**Published:** 2025-02-08

**Authors:** Xueshan Sun, Zhongliang Zhou, Wenhua Wang

**Affiliations:** School of Public Policy and Administration, Xi’an Jiaotong University, Xi’an 710049, China; sunxueshan@xjtu.edu.cn

**Keywords:** medical errors, quality of care, confidence, physicians, primary care system

## Abstract

**Background/Objectives:** Patient safety is fundamental to primary healthcare, and medical errors impose a considerable burden on patients globally. However, the impact of medical errors on primary healthcare physicians remains understudied, especially in developing countries. This study aimed to examine the associations between self-reported medical errors and physicians’ performance and confidence in Chinese primary care practice. **Methods:** A cross-sectional survey was conducted from November 2021 to May 2022 with 224 primary care physicians from 38 community health centers (CHCs) across four large cities in China. The quality of clinical and preventative care, and confidence in managing commonly occurring diseases, multimorbidity, and common mental health disorders served as indicators of performance and confidence, respectively. Hierarchical linear regression and linear regression with cluster-robust standard errors were employed. **Results:** Clinical care quality (*β* = −0.159, SE = 0.075, *p* < 0.05), preventive care quality (*β* = −0.165, SE = 0.068, *p* < 0.05), confidence in managing multimorbidity (*β* = −0.175, SE = 0.074, *p* < 0.05), and confidence in managing common mental health disorders (*β* = −0.189, SE = 0.076, *p* < 0.05) were negatively associated with self-reported medical errors, with scores of 4.08 (SD 0.95), 3.59 (SD 0.87), 3.63 (SD 1.04), and 3.10 (SD 1.21) out of 5 (where 5 represents the best possible score), respectively. The association between self-reported medical errors and confidence in managing commonly occurring diseases (*β* = −0.063, SE = 0.075, *p* > 0.05) was not statistically significant, with a score of 3.81 (SD 1.00) out of 5 (where 5 represents the best possible score). **Conclusions:** This study offers new insight into the associations between self-reported medical errors and primary healthcare physicians’ performance and confidence. It is crucial for CHCs to be aware of the impact of self-reported medical errors on physicians’ performance in delivering clinic and preventative care, and confidence in managing multimorbidity and common mental health disorders. Strategies such as strengthening organizational support should be developed to maintain performance and rebuild confidence in delivering care for physicians who were involved in medical errors.

## 1. Introduction

Medical errors are defined as “the failure to complete a planned action as intended or the use of a wrong plan to achieve an aim” by the Institution of Medicine (IOM) [1]. The term “medical errors” is an umbrella that refers to all errors that occur in a healthcare system, which generally include medication errors, equipment failures, diagnostic errors, and laboratory errors [2,3]. According to the seminal 1999 IOM report, between 44,000 and 98,000 patients die annually because of medical errors in the United States [4]. Experts estimated that more people die from medical errors in hospitals than from motor vehicle accidents, breast cancer, or acquired immunodeficiency syndrome [5]. The economics of medical errors has received great attention, and a study reported that medical errors may have cost the United States USD 19.5 billion in 2008 [6]. Additionally, many risk factors have been proven to contribute to the occurrence of medical errors [2,7,8], and medical errors are sometimes unavoidable.

Primary healthcare institutions serve as “gatekeepers” for public health [9]. Primary healthcare institutions are increasingly responsible for managing both common diseases and chronic diseases, particularly multimorbidity, due to evolving societal and disease trends [10]. Additionally, the World Health Organization (WHO) has called for the integration of mental health into primary care [11]. In order to better leverage their role in treating and managing common and frequently occurring diseases, chronic diseases, and mental health disorders, high-quality healthcare is the core of primary healthcare institutions [12]. Meanwhile, as frontline providers in the primary healthcare system who directly interact with patients, physicians require confidence in treating and managing diseases. This confidence is important, it reflects their level of expertise and helps them maintain optimal professional performance [13]. Especially when dealing with patients with complex conditions, facing risks, and under professional pressures, physicians require even stronger confidence to ensure the delivery of high-quality healthcare.

Ensuring the quality of primary healthcare and physicians’ confidence in delivering care is crucial for primary healthcare institutions to fulfill their role as gatekeepers. Various factors have been reported to influence physicians’ performance and confidence in providing primary healthcare services. Organizationally, organizational support, culture, equipment, and structure have been linked to the quality of care and physicians’ confidence [14,15,16]. Individually, professional fulfillment, psychological safety, and organizational citizenship behavior are associated with the quality of care and physicians’ confidence [14,15]. In addition to these factors, experience with medical errors has also been linked to physicians’ performance and confidence. There is evidence reporting a correlation between medical errors and physician burnout, which has been associated with poor-quality care delivered by health professionals [17]. Another survey reported a significant correlation between pediatric nurses’ experience with medical errors and their self-efficacy and competence [18]. Medical errors significantly impact the involved patients and healthcare providers [19,20,21]. In recent years, the issue of patient safety arising from medical errors has garnered widespread attention, prompting numerous studies to reduce medical errors and enhance patient safety [22,23,24,25]. Exploring the impact of medical errors on physicians is critically important, as experience with medical errors may adversely affect their performance in delivering care and diminish their confidence in primary healthcare practice. However, compared with the increasing number of studies exploring the impact of medical errors on patients, fewer studies have explored the impact of medical errors on physicians, especially in primary healthcare centers in developing countries. In fact, some physicians who were involved in medical errors were reported to become the “second victims” of these events [4]. As the first point of contact for patients in many communities, primary healthcare physicians play a crucial role in patient safety [21].

To further explore the association between experience with medical errors and physicians’ performance and confidence in delivering primary care, behavioral economics theory provides a reference. Behavioral economics theory states that physicians’ behaviors may be affected by their previous experience and defines a framework that has been increasingly applied in the study of physicians’ behavior in recent years [26]. This theory suggests that negative events such as medical errors and medical disputes can affect physicians’ psychological and behavioral states [27]. Studies have validated that behavioral economics theory can be used to explain physicians’ behavior and guide the exploration of pathways influencing physicians’ behavior [27,28]. Therefore, based on the behavioral economics theory, we hypothesize that there are significant associations between physicians’ experience with medical errors and their performance and confidence in delivering primary care, even after controlling for individual-level and organizational-level variables. This study aims to offer new insights into health policies designed to build a safer primary healthcare system and to enhance primary care physicians’ performance and confidence after medical errors.

## 2. Methods

### 2.1. Data Collection

A cross-sectional study was conducted at 38 community health centers (CHCs) located in four large cities in China between November 2021 and May 2022. CHCs in Shanghai (10 CHCs), Shenzhen (14 CHCs), Tianjin (eight CHCs), and Jinan (six CHCs) were surveyed using a convenience sampling method. The four cities were selected for two main reasons: first, the four cities are larger, developed, and located in the south and north of China, strengthening the representativeness of the samples; second, the support of health bureaus can guarantee the feasibility of the study. In each CHC, a physician was included if they were an employee of one of the 38 CHCs and were on duty on the day of the survey, regardless of their age, gender, etc. A physician was excluded if they refused to participate in the questionnaire. Face-to-face questionnaire surveys were conducted. A self-administered questionnaire was used, which included the physician’s sociodemographic characteristics, organizational characteristics, job characteristics, self-reported medical errors, quality of clinical and preventative care, and confidence in delivering primary care. The sample size of physicians was calculated according to the requirements proposed for multilevel regression models [16,29,30,31]. Rigorous data quality control was implemented during the investigation. Before the survey, the investigators (Z.Z. and W.W.) underwent training that covered the questionnaire’s context, survey procedures, methods for addressing potential questions posed by physicians, and techniques to ensure the completeness and logical coherence of their responses. Only when they qualified to conduct the survey were they allowed to continue participating. During the survey, missing or wrong answers were identified, and the participants were asked again. After the survey, the double-data entry method was used for accuracy.

Finally, a total of 224 physicians working in 38 CHCs were enrolled, with a 100% response rate. Written informed consent was obtained from all participants, and the questionnaires were anonymous. Ethical approval was obtained from the Biomedical Ethics Committee of the Medical Department of the authors’ institute (No. 2020-1344).

### 2.2. Measures

#### 2.2.1. Self-Reported Medical Errors

Self-reported medical errors were identified as a key predictor and were measured using a translated scale proposed by Trockel [32]. Four items were measured to calculate physicians’ self-reported medical errors from the perspective of those that did result in or could have resulted in patient harm, medication errors, and laboratory test errors by responses to questions such as “I made a medical error that did result in patient harm”. The responses were scored on a Likert scale ranging from 1 to 6, where 1 was in the last week, 2 was in the last month, 3 was in the last 3 months, 4 was in the last year, 5 was in my lifetime, and 6 was never. The score of each item was categorized into a binary response, where 0 was never had an experience with self-reported medical errors, and 1 indicated an experience with medical errors. The total scores on the four items were used to indicate the level of the physician’s experience with self-reported medical errors. Cronbach’s α for the self-reported medical errors scale was 0.922 in this study.

#### 2.2.2. Quality of Clinical Care

In line with previous study [33], the quality of clinical care was measured using a single item with a five-point response: “Do you often use evidence-based treatment guidelines released by national or medical associations to treat patients with chronic diseases” (1 = never to 5 = always).

#### 2.2.3. Quality of Preventative Care

The quality of preventative care was measured using the modified and translated preventive services for health behaviors scale proposed by Hung [34]. Physicians’ behaviors regarding the delivery of preventative care were measured as health risk assessments, referral to community programs, individual health counseling, and group health counseling related to patient health behaviors, including smoking, drinking, dieting, and exercising. A five-point response was used, with scores ranging from 1 for never to 5 for always. The average scores of the four items were used to indicate the physician’s quality of preventive care. Cronbach’s α for the quality of the preventive scale was 0.855 in this study.

#### 2.2.4. Confidence in Delivering Primary Care

In global consensus, the current primary healthcare system emphasizes not merely the treatment of commonly occurring diseases, but equally importantly, the management of multimorbidity and common mental health disorders, which have consistently ranked as key priorities in primary healthcare [35,36]. Therefore, in accordance with previous studies [15,31], three items were used to gauge physicians’ confidence in managing commonly occurring diseases, multimorbidity, and common mental health disorders. “How confident are you in providing optimal healthcare for patients with commonly occurring diseases/multimorbidity/common mental health disorders?”. A five-point response was adopted, with scores ranging from 1 for no confidence to 5 for fully confident. The score of each item was used to indicate the three dimensions of confidence.

#### 2.2.5. Covariates

Control variables include both the physician-level and organization-level variables. Variables at the physician level include: (1) age (continuous variable); (2) sex (male = 0, female = 1); (3) marital status (1 = single, 2 = married/cohabiting, 3 = divorced/widowed); (4) education level (1 = high school or below, 2 = undergraduate/college, 3 = master or above); (5) tenured position (0 = do not have tenured position, 1 = have tenured position); (6) years of working experience (continuous variable); (7) medical specialty (1 = clinical medicine, 2 = public health, 3 = traditional Chinese medicine/Integrated Chinese and Western medicine, 4 = other specialties in medicine); (8) burnout. Burnout was measured using the brief instrument of burnout in physicians proposed by Trockel [32]. A five-point scale was used, and four items were included in the instrument. The average score of the four items was used.

Organization-level covariates include: (1) organizational ownership (0 = government-managed, 1 = public hospital-managed); (2) accreditation status (accredited by national or provincial health authorities or not, 0 = not accredited, 1 = accredited); (3) organizational size (continuous variable), measured by number of staff in each CHC; (4) information technology functional capacity. Organizational information technology functional capacity was measured by the 14-item checklist proposed by Davis K [33]. The answer is binary (0 = no and 1 = yes), and the scores of information technology functional capacity were categorized into three levels according to the total scores: low level ≤ 2, 3 ≤ middle level ≤ 6, and high level ≥ 7 in this study.

### 2.3. Analytical Strategy

First, the descriptive statistics method was used to describe the basic characteristics of included physicians and CHCs. Numbers and percentages were used to report categorical variables, mean and standard deviation were used for continuous variables. Second, Spearman’s rank correlation coefficient was used to examine the correlation between the study variables. Finally, because the physician-level variables were nested within the organization-level variables, the intraclass correlation coefficient (ICC) tested whether the hierarchical linear regression models could be adapted. ICC can reflect the extent to which variation in the outcome variables can be explained by organizational-level factors. The threshold of ICC suggested by Cohen [29] was 0.059. The ICC values of clinical care quality, prevention care quality, confidence in managing commonly occurring diseases, confidence in managing multimorbidity, and confidence in managing common mental health disorders were 0.086, 0.145, 0.099, 0.028, and 0.021, respectively. The values of ICC for confidence in managing multimorbidity and confidence in managing common mental health disorders were lower than 0.059, indicating that the variation in the two variables can be explained within CHCs rather than between CHCs; therefore, the linear regression models with cluster-robust standard errors were estimated. The values of ICC for clinical care quality, confidence in managing commonly occurring diseases, and prevention care quality were greater than 0.059, and multilevel regression models were estimated. Collinearity was measured by calculating the variance inflation factor (VIF) value, and the VIF value in this study was less than 5. A value of *p* < 0.05 was considered significant in this study. All analyses were conducted using Stata 18.0.

## 3. Results

### 3.1. Characteristics of Physicians and CHCs

The characteristics of the 224 physicians and 38 CHCs included in the study are shown in Table 1. Of the 224 physicians, 40.18% were male, with a mean age of 36.82 years (SD 7.76). Of them, 77.68% were married or cohabiting, only 1.79% were divorced or widowed, 79.91% had undergraduate or college degrees, and 16.07% had master’s degrees or above. The average working experience was 10.81 years (SD 8.04); 75.89% had tenured positions, and 66.07% specialized in clinical medicine. The average burnout score was 2.54 out of 5 (SD 1.02). Of the 38 CHCs, 60.53% were government-managed, and 57.89% were accredited by national or provincial health authorities. The mean size was determined to be 30.24 (SD 30.02) using the number of all staff to measure organizational size. Of the CHCs, 21.05% reported low and middle-level information technology functional capacity.

### 3.2. Scores and Reliability of the Study Variables

As shown in Table 2, the mean scores for major self-reported medical errors that could have resulted in harm, self-reported medical errors that did result in harm, and medication errors and laboratory test errors were 5.92 (SD 0.46), 5.91 (SD 0.48), 5.87 (SD 0.49), and 5.88 (SD 0.49) out of 6, respectively. Among the five outcome variables, the highest score was reported for clinical care quality, which was 4.08 out of 5 (SD 0.95). For the preventive care quality scores, the average score was 3.59 out of 5 (SD 0.87), with the highest score for referral to community program services (3.76 out of 5) and the lowest score for risk assessments (3.33 out of 5). The scores for physicians’ confidence in delivering optimal healthcare for patients with commonly occurring diseases, multimorbidity, and common mental health disorders were 3.81 (SD 1.00), 3.63 (SD 1.04), and 3.10 (SD 1.21), respectively.

### 3.3. Correlations of the Study Variables

The correlation matrix for self-reported medical errors and the five outcome variables is shown in Table 3. The findings showed that physicians’ experience with self-reported medical errors was significantly negatively associated with clinical care quality (r = −0.1816, *p* < 0.05) and their confidence in managing common mental health disorders (r = −0.1381, *p* < 0.05), indicating that experience with medical errors may decrease physicians’ clinical care quality and confidence in managing common mental health disorders.

### 3.4. Association Between Physicians’ Self-Reported Medical Errors, Performance, and Confidence in Delivering Primary Care

The results of the linear models are shown in Table 4. The results demonstrated that physicians’ experience with self-reported medical errors was significantly negatively associated with clinical care quality (*β* = −0.159, SE = 0.075, *p* < 0.05), preventive care quality (*β* = −0.165, SE = 0.068, *p* < 0.05), confidence in managing multimorbidity (*β* = −0.175, SE = 0.074, *p* < 0.05), and confidence in managing common mental health disorders (*β* = −0.189, SE = 0.076, *p* < 0.05). The association between physicians’ experience with self-reported medical errors was not significantly associated with their confidence in managing commonly occurring diseases (*β* = −0.063, SE = 0.075, *p* > 0.05).

Among the physician-level control variables, physicians with master’s degrees and above showed lower degrees of confidence in managing common mental health disorders than physicians with high school or below (*β* = −0.823, SE = 0.406, *p* < 0.05). Physicians whose specialty was traditional Chinese medicine/Integrated Chinese and Western medicine showed lower quality of clinical care (*β* = −0.375, SE = 0.165, *p* < 0.05) than physicians whose specialty was clinical medicine. Physicians whose specialty was public health and preventive medicine showed lower degrees of confidence in managing commonly occurring diseases (*β* = −0.513, SE = 0.253, *p* < 0.05). Physician burnout was negatively associated with clinical care quality (*β* = −0.134, SE = 0.057, *p* < 0.05), preventive care quality (*β* = −0.172, SE = 0.052, *p* < 0.05), confidence in managing common occurring diseases (*β* = −0.281, SE = 0.058, *p* < 0.001), confidence in managing multimorbidity (*β* = −0.311, SE = 0.073, *p* < 0.001), and confidence in managing common mental health disorders (*β* = −0.292, SE = 0.086, *p* < 0.05). Among the organization-level control variables, physicians at CHCs with 36–55 and 56–100 staff reported significantly lower degrees of confidence in managing commonly occurring diseases (*β* = −0.449, SE = 0.224, *p* < 0.05) and lower quality of preventive care delivery (*β* = −0.690, SE = 0.250, *p* < 0.05) than physicians at CHCs with organizational sizes of ≤35. Physicians at CHCs with higher levels of information technology functional capacity showed higher levels of clinical care quality (*β* = 0.364, SE = 0.168, *p* < 0.05) and lower degrees of confidence in managing common mental health disorders (*β* = −0.462, SE = 0.156, *p* < 0.05) than physicians at CHCs with low information technology functional capacity.

Akaike information criterion (AIC) values were calculated for both the null models and full models for the outcome variables of clinical care quality, preventive care quality, and confidence in common occurring diseases. All AIC values for the full models were lower than those of the null models, indicating that incorporating physicians’ self-reported medical errors and covariables could enhance the fit of the hierarchical linear models in this study.

The association between each item on the self-reported medical error instrument and the five outcome variables is shown in Table 5. With all covariables controlled, significant associations were found between the first item (“I made a major medical error that could have resulted in patient harm”) and clinical care quality, preventive care quality, confidence in managing multimorbidity, and confidence in managing common mental health disorders. The second item (“I made a medical error that did result in patient harm”) was significantly associated with clinical care quality, confidence in managing multimorbidity, and confidence in managing common mental health disorders. The third item (“I ordered the wrong medication”) was not significantly associated with any outcome variables. The fourth item (“I ordered the wrong laboratory test”) was significantly negatively associated with preventive care quality.

## 4. Discussion

This study conducted a survey of 224 physicians in 38 CHCs in four large cities in China to examine the potential impact of primary care physicians’ experience with self-reported medical errors on their performance and confidence in delivering primary care. The study results showed that physicians’ self-reported medical errors were negatively associated with clinical care quality, preventive care quality, confidence in managing multimorbidity, and common mental health disorders. However, physicians’ experience with self-reported medical errors was not associated with their confidence in managing commonly occurring diseases.

Primary care should be first-contact, comprehensive, coordinated, continuous, and patient-centered to satisfy health needs, and the delivery of clinical care and preventive care are essential elements of primary care [14]. Different methods are used to measure primary care quality, including patient-perceived quality, standardized patient measurements, and physicians’ self-reported quality [37]. A physicians’ self-report method was appropriate to explore the impact of experience with medical errors on their performance and confidence in this study. Previous studies reported that diverse influencing factors may affect physicians’ performance in delivering primary care. First, evidence supports a significant association between physicians’ self-reported primary care quality and organizational citizenship behavior, professional fulfillment, organizational structure and support, and organizational culture [14]. Second, region and patient loyalty were also found to be factors influencing patient-perceived care quality [38]. Third, clinician patterns, registered nurse ratio in the practice, and the number of care hours were also reported to be factors influencing the quality of care [39,40]. Accusations of medical malpractice are a massive source of physician stress following medical errors and may cause the development of medical malpractice stress syndrome (MMSS) [41]. MMSS may result in suboptimal physician performance and lead to unnecessary ordered examinations, delayed therapeutic processes, or retirement among healthcare professionals [41]. This study also confirmed that physicians’ experience with self-reported medical errors was negatively associated with their performance in delivering both clinic and preventive care. The findings could guide the development of strategies to improve their performance in delivering primary care, especially for physicians who reported experience with medical errors.

Medical errors may also translate into different adverse physical and psychological symptoms among physicians, such as anger, frustration, anxiety, sleeping disorders, depression, and especially loss of confidence, which should be a major concern to healthcare organizations [41,42]. Previous studies reported different conclusions regarding the relationship between medical errors and physicians’ loss of confidence. On the one hand, the adverse impact of experience with medical errors on physicians’ confidence in managing diseases was reported. A survey of physicians who had experience with medical errors found that 44% of physicians reported a loss of confidence, and 61% reported increased anxiety about future errors [43]. The adverse impact was also confirmed in this study; physicians’ experience with medical errors was negatively associated with their confidence in managing multimorbidity and common mental health disorders. On the other hand, no significant association was found between medical errors and physicians’ confidence in managing commonly occurring diseases in this study, consistent with another studies. For example, junior doctors were reported to be aware that they were making prescribing errors; however, they also reported a high level of confidence in all aspects of prescribing [44]. Similarly, another study reported that the relationship between confidence in prescribing and the awareness of prescribing errors was not significant among medical interns [45]. Physicians’ confidence was divided into three types based on disease categories in the present study, leading to the conclusion that medical errors had inconsistent impacts on different types of confidence. Currently, mental health disorders and multimorbidity are becoming increasingly severe globally, and therefore there is a consensus that primary healthcare should undertake more responsibilities in delivering mental health and multimorbidity care [10,46]. Our findings also demonstrate that it is crucial for community healthcare centers to be aware of the impact of medical errors on physicians’ confidence in managing mental health disorders and multimorbidity, so as to more effectively integrate mental health and multimorbidity services into primary healthcare.

Medical errors were measured in this study by four behaviors: making major medical errors that could have resulted in patient harm, medical errors that did result in patient harm, wrong medications, and wrong laboratory tests. Physicians’ behaviors in making a major medical error that could have resulted in patient harm or did result in patient harm were more negatively associated with physicians’ performance and confidence than the behavior of prescribing the wrong medication or ordering the wrong laboratory test. This finding may be attributed to the different levels of emotional impact caused by different severities of medical errors. A previous study reported that the top four reported emotional responses were frustration, embarrassment, anger, and guilt, and an emotional response was reported more often when there was a possibility of harm [47]. Adverse emotions may also contribute to physicians’ loss of confidence and suboptimal patient care performance [43,48]. However, another study reported that incidents with minor or no harm still invoked emotional responses from family physicians and their office staff, suggesting that it is necessary to understand the potential impact mechanisms of wrong medications and laboratory tests on physicians’ performance and confidence in future studies [47].

In terms of covariates, physician burnout, specialty, education level, organizational size, and level of information technology functional capacity were found to be significantly associated with physicians’ behavior in delivering primary care. Physician burnout is prevalent internationally and is defined as a work-related syndrome involving emotional exhaustion, a sense of reduced personal accomplishment, and depersonalization [49]. Physician burnout has been linked to adverse effects both on physicians and patients [50,51]. This study also confirmed that burnout was negatively associated with physicians’ performance and confidence in delivering primary care. Diverse drivers of physician burnout have been reported, such as inefficient work processes, work–home conflicts, and organizational support [49]. When implementing strategies to improve medical errors affecting physicians’ performance and confidence, CHCs should also invest more time and effort in implementing strategies to mitigate physician burnout. This study found that higher technology functional capacity predicted better clinical care quality, whereas a contradictory finding was found between technology functional capacity and physicians’ confidence in common mental health disorders. This finding was consistent with a previous literature review, in which 92% of the included studies reported a positive impact of health information technology on quality, efficiency, and provider satisfaction in healthcare delivery [52]. In general, larger CHCs are usually visited by more patients. A previous study reported that increasing panel patient size was associated with small decreases in cancer screening, continuity, and comprehensiveness [53]. This study also confirmed that larger organizational size was negatively associated with preventative care quality and physicians’ confidence in commonly occurring diseases in this study, consistent with the previous findings.

Since significant associations were found between physicians’ experience with self-reported medical errors and lower quality of care and lower degrees of confidence, directors of CHCs or related health policymakers should provide support for physicians who report medical errors to maintain their role in delivering primary care and rebuild confidence. Medical errors are inevitable for most physicians. In some countries, there exist “no-fault” systems, such as the US communication-and-resolution programs and New Zealand’s no-fault administrative compensation scheme [54]. However, China currently lacks such system. Therefore, based on the findings of this study, health policymakers and CHCs need to collaborate to help physicians mitigate the negative impacts of medical errors, especially in similar Chinese settings for global primary healthcare systems. First, organizational support resources to support physicians after medical errors should be provided, as one study reported that physicians would feel a variety of adverse emotions after medical errors, and have urgent desires to obtain organizational and individual support to reduce the negative impact of adverse emotions [21]. Second, multidisciplinary actions that can teach physicians how to better cope with medical error experiences, including improvements in work environmental factors and stress management programs, can be implemented to help them self-manage the adverse impact of medical errors [55]. Third, patients and their families are the direct “victims” of medical errors, and positive and proactive patient–physician communication training must be provided for all physicians. Moreover, policymakers also need to guide patients in correctly managing medical errors, helping to prevent the negative impact of medical errors from further expanding [56].

The findings in this study should be interpreted with caution because of several limitations. First, because of the cross-sectional study design, the results can only indicate associations between physicians’ experience with medical errors and the delivery of primary care. No causal inference can be obtained. Second, self-administered questionnaires and a convenience sampling method were used to measure the variables. While strict quality control was conducted during the entire investigation, recall bias and selection bias may still exist. Third, although important physician-level and organization-level control variables that were reported in previous studies were included in this study, other potential confounding factors, such as in-service training program activities, physicians’ satisfaction scores, the complexity of patient cases, patient demographics, blame-free and non-punitive culture for medical errors, and leadership support at an organization-level, could influence the association between primary care physicians’ self-reported medical errors and the delivery of care. Fourth, as the survey was conducted during the COVID-19 pandemic, the restricted sample size and potential impacts of the pandemic on physicians’ confidence should be considered when interpreting findings in this study. Given these limitations, we recommend further research to explore the impact of medical errors on physicians, as well as their impact on nursing staff. Additionally, longitudinal studies incorporating potential covariates are needed to better understand how experiencing medical errors affects healthcare professionals.

## 5. Conclusions

We investigated physicians’ self-reported medical errors in community healthcare centers and their associations with physicians’ performance and confidence in delivering care. Physicians’ self-reported medical errors were negatively associated with clinical and preventive care quality and confidence in managing multimorbidity and common mental health disorders but were not associated with their confidence in managing commonly occurring diseases. As the findings from this study indicate, experiencing medical errors can be associated with physicians’ performance and confidence in delivering care, and psychological factors may contribute to these associations. We call for attention to be paid to the confidence levels and performance of primary healthcare physicians involved in medical errors. More importantly, there is a need for health policymakers and community healthcare centers to collaborate to mitigate the negative impacts of medical errors on physicians. For example, psychological support resources, multidisciplinary interventions, and communication training can be provided to physicians who have experienced medical errors.

## Figures and Tables

**Table 1:** Characteristics of physicians and CHCs.

Characteristics	Number (%)
**Physician level (N = 224)**	
Age, mean (SD)	36.82 (7.76)
Sex, n (%)	
Male	90 (40.18)
Female	134 (59.82)
Marital status, n (%)	
Single	46 (20.54)
Married/Cohabiting	174 (77.68)
Divorced/Widowed	4 (1.79)
Education level, n (%)	
High school or below	9 (4.02)
Undergraduate/college	179 (79.91)
Master or above	36 (16.07)
Having tenured position, n (%)	170 (75.89)
Years of working experience, mean (SD)	10.81 (8.39)
Medical specialty, n (%)	
Clinical medicine	148 (66.07)
Public health and preventive medicine	21 (9.38)
Traditional Chinese medicine/Integrated Chinese and Western medicine	37 (16.52)
Other specialties in medicine	18 (8.04)
Burnout	2.54 (1.02)
**Organizational level (N = 38)**	
Organizational ownership, n (%)	
Government-managed	23 (60.53)
Public hospital-managed	15 (39.47)
Accredited status, n (%)	22 (57.89)
Organizational size ^a^	30.24 (30.02)
≤35	13 (34.21)
36–55	7 (18.42)
56–100	7 (18.42)
>100	11 (28.95)
Information technology functional capacity, n (%)	
Low and middle level	8 (21.05)
High level	30 (78.95)

PCPs—primary care physicians; CHCs—community health centers; SD—standard deviation. ^a^ The number of all staff was measured as the organizational size.

**Table 2:** Scores and items of physicians’ self-reported medical errors, quality of care, and confidence in delivering primary care.

Variables	Measurement Items	Response Categories	Mean	SD	Cronbach α
Self-reported medical error experience	I made a major medical error that could have resulted in patient harm	Likert 6-point scale (1 = in the last week to 6 = never)	5.92	0.46	0.922
	I made a medical error that did result in patient harm		5.91	0.48	
	I ordered the wrong medication		5.87	0.49	
	I ordered the wrong laboratory test		5.88	0.49	
Clinical care quality	Do you often use ‘evidence-based’ treatment guidelines released by national or medical associations to treat patients with chronic diseases?	Likert 5-point scale (1 = never to 5 = always)	4.08	0.95	--
Preventive care quality	How often do you use a health risk assessment questionnaire to identify patients who may benefit from counseling for the followings (smoking, alcohol, diet, exercise)?	Likert 5-point scale (1 = never to 5 = always)	3.33	1.18	0.855
	How often do you refer your patients to community programs (counseling/support groups) for the followings (smoking, alcohol, diet, exercise)?		3.76	0.96	
	How often do you use nurses/health educators within the CHC for individual counseling to your patients with the following (smoking, alcohol, diet, exercise)?		3.61	1.00	
	How often do you use group counseling activities for patients with the following (smoking, alcohol, diet, exercise)?		3.64	1.03	
Confidence in managing commonly occurring diseases	How confident are you in providing optimal healthcare for patients with commonly occurring diseases?	Likert 5-point scale (1 = no confidence to 5 = fully confident)	3.81	1.00	--
Confidence in managing multimorbidity	How confident are you in providing optimal healthcare for patients with multimorbidity?		3.63	1.04	--
Confidence in managing common mental health disorders	How confident are you in providing optimal healthcare for patients with common mental health disorders?		3.10	1.21	--

Note. SD—standard deviation; CHC—community health center.

**Table 3:** Correlation matrix of the main variables.

Variables	SME	CCQ	PCQ	CCO	CMM	CMH
SME	1.0000					
CCQ	−0.1816 **	1.0000				
PCQ	−0.0969	0.4736 ***	1.0000			
CCO	−0.0333	0.3807 ***	0.4704 ***	1.0000		
CMM	−0.1233 *	0.4285 ***	0.5551 ***	0.8283 ***	1.0000	
CMH	−0.1381 **	0.2575 ***	0.4520 ***	0.5683 ***	0.6417 ***	1.0000

Note. *** *p* < 0.01, ** *p* < 0.05, * *p* < 0.1; SME—self-reported medical errors; CCQ—clinical care quality; PCQ—preventive care quality; CCO—confidence in managing common occurring diseases; CMM—confidence in managing multimorbidity; CMH—confidence in managing mental health disorders.

**Table 4:** Linear regression models examining the role of physicians’ self-reported medical errors in quality of care and confidence in delivering primary care.

Variables	CCQ ^a^		PCQ ^a^		CCO ^a^		CMM ^b^		CMH ^b^	
	*β*	SE	*β*	SE	*β*	SE	*β*	SE	*β*	SE
**Key predictor**										
Self-reported medical errors	−0.159 **	0.075	−0.165 **	0.068	−0.063	0.075	−0.175 **	0.074	−0.189 **	0.076
**Physician-level covariates**										
Age	0.014	0.016	0.001	0.014	0.024	0.016	0.021	0.017	0.026	0.023
Gender (ref = male)										
Female	−0.226 *	0.120	−0.006	0.108	−0.151	0.120	−0.049	0.118	−0.002	0.165
Marital status (ref = single)										
Married/Cohabiting	−0.045	0.183	0.011	0.165	−0.100	0.183	0.025	0.210	0.211	0.267
Divorced/Widowed	−0.091	0.477	0.026	0.432	−0.038	0.479	0.243	0.425	0.226	0.531
Education level (ref = high school or below)										
Undergraduate/college	−0.274	0.302	−0.440	0.272	0.054	0.303	−0.094	0.247	−0.570	0.358
Master or above	−0.187	0.354	−0.434	0.322	−0.032	0.357	−0.156	0.316	−0.823 **	0.406
Having tenured position (ref = no)										
Having tenured position	−0.138	0.177	−0.050	0.159	0.161	0.177	0.201	0.196	−0.163	0.269
Years of working experience	0.011	0.014	0.013	0.013	0.007	0.014	0.004	0.015	−0.004	0.023
Medical specialty (ref = clinical medicine)										
Public health and preventive medicine	−0.267	0.253	−0.143	0.228	−0.513 **	0.253	−0.465	0.327	0.390	0.411
Traditional Chinese medicine/Integrated Chinese and Western medicine	−0.375 **	0.165	−0.257 *	0.149	−0.260	0.165	−0.141	0.188	0.082	0.201
Other specialties in medicine	0.121	0.231	0.034	0.210	0.070	0.233	−0.033	0.187	0.409	0.276
Burnout	−0.134 **	0.057	−0.172 **	0.052	−0.281 ***	0.058	−0.311 ***	0.073	−0.292 **	0.086
**Organizational-level covariates**										
Organizational ownership (ref = government-managed)										
Public hospital-managed	−0.292	0.200	−0.186	0.205	0.287	0.215	0.213	0.164	0.080	0.221
Accredited status (ref = not accredited)										
Accredited	−0.023	0.153	−0.161	0.159	−0.253	0.166	−0.240	0.151	−0.102	0.162
Organizational size (ref = size ≤ 35)										
36–55	−0.225	0.208	−0.390 *	0.214	−0.449 **	0.224	−0.310	0.184	−0.290	0.277
56–100	−0.351	0.241	−0.690 **	0.250	−0.261	0.261	−0.349	0.264	−0.381	0.296
>100	−0.395 *	0.220	−0.445 *	0.230	−0.080	0.239	−0.165	0.195	−0.209	0.258
Information technology functional capacity (ref = low and middle level)										
High level	0.364 **	0.168	0.032	0.175	0.073	0.182	−0.048	0.127	−0.462 **	0.156
ICC	0.086		0.145		0.099		0.028		0.021	
AIC	605.934 (613.244)		565.225 (569.567)		609.687 (635.194)		631.643		715.230	

Note. *** *p* < 0.001, ** *p* < 0.05, * *p* < 0.1; *β*—coefficient; SE—standard error; ICC—intraclass correlation coefficient; AIC—Akaike information criteria, and the values in the parentheses are the AIC values of the null models; SME—self-reported medical errors; CCQ—clinical care quality; PCQ—preventive care quality; CCO—confidence in managing common occurring diseases; CMM—confidence in managing multimorbidity; CMH—confidence in managing mental health disorders. ^a^ because the values of ICC of clinical care quality, preventive care quality and confidence in managing common occurring diseases were higher than 0.059, the hierarchical linear regression models were conducted. ^b^ because the values of ICC of confidence in managing multimorbidity and confidence in managing mental health disorders were lower than 0.059, the linear regression models with cluster-robust standard errors were conducted.

**Table 5:** Linear regression models examining the association of four domains of physicians’ self-reported medical errors with quality of care and confidence in delivering primary care.

Domains	CCQ		PCQ		CCO		CMM		CMH	
	*β*	SE	*β*	SE	*β*	SE	*β*	SE	*β*	SE
Item 1	−0.605 **	0.294	−0.612 **	0.267	−0.494 *	0.294	−0.480 **	0.224	−0.580 **	0.257
Item 2	−0.631 **	0.262	−0.277	0.240	−0.341	0.263	−0.652 **	0.203	−0.526 **	0.233
Item 3	−0.205	0.206	−0.281	0.187	0.044	0.206	−0.332	0.237	−0.435 *	0.242
Item 4	−0.324	0.215	−0.524 **	0.193	−0.076	0.215	−0.361	0.251	−0.391	0.270

Note. ** *p* < 0.05, * *p* < 0.1; Item 1 = “I made a major medical error that could have resulted in patient harm”; Item 2 = “I made a medical error that did result in patient harm”; Item 3 = “I ordered the wrong medication”; Item 4 = “I ordered the wrong laboratory test”; SME—self-reported medical errors; CCQ—clinical care quality; PCQ—preventive care quality; CCO—confidence in managing common occurring diseases; CMM—confidence in managing multimorbidity; CMH—confidence in managing mental health disorders. The same control variables as in Table 4 were included in the models.

## Data Availability

The data underlying this study can be shared on reasonable request to the corresponding author.

## Abbreviations

The following abbreviations are used in this manuscript:

IOM	Institution of Medicine
WHO	World Health Organization
CHCs	community health centers
ICC	intraclass correlation coefficient
VIF	variance inflation factor
AIC	Akaike information criterion
MMSS	medical malpractice stress syndrome
